# Study on the Influences of Adding Rare Earth Ce on the Precipitation Behaviors of TiN Inclusions in 20CrMnTi

**DOI:** 10.3390/ma16165598

**Published:** 2023-08-12

**Authors:** Jian Wang, Jun Peng, Lixia Liu, Fang Zhang, Jihua Peng, Hao Tang, Jie Zheng, Shengli An

**Affiliations:** 1 School of Materials and Metallurgy, Inner Mongolia University of Science and Technology, Baotou 014010, China; jianwang.liaoning@163.com (J.W.); zhangfang2006322@163.com (F.Z.);; 2 Inner Mongolia Key Laboratory of Advanced Ceramic Materials and Devices, Inner Mongolia University of Science and Technology, Baotou 014010, China; 3 Key Laboratory of Green Extraction & Efficient Utilization of Light Rare-Earth Resources, Ministry of Education, Inner Mongolia University of Science and Technology, Baotou 014010, China; 4 School of Rare Earth Research and Development, Inner Mongolia University of Science and Technology, Baotou 014010, China

**Keywords:** gear steel, TiN inclusions, rare earth Ce, precipitation

## Abstract

The morphologies and sizes of TiN inclusions in gear steel 20CrMnTi have a significant impact on its service performance. This paper selects rare earth Ce to modify TiN inclusions in 20CrMnTi. The inclusions are analyzed by SEM (scanning electron microscope), EBSD (electron back-scattered diffraction), EDS (energy disperse spectroscopy), and OTS statistical software, and Thermo-Calc software is used to calculate the inclusion formations. The inclusions of MgAlO_4_-Ce and CeAlO_3_ can be formed when rare earth Ce is added into 20CrMnTi, which becomes TiN nucleation core after precipitation. Without the addition of rare earth Ce, square TiN inclusions ranging from 2 to 5 μm account for 60% of the total inclusions in 20CrMnTi. After adding rare earth Ce, the TiN inclusions in 20CrMnTiCe account for 36.7% of the total inclusions. Due to the new phase formations of MgAlO_4_-TiN and CeAlO_3_-TiN with sizes less than 2 μm, the titanium-containing inclusions are refined. Fatigue tests are conducted on the steels before and after the addition of Ce. The average fatigue lives of 20CrMnTi do not reach 10^7^ times, and the deviations between the maximum and minimum fatigue lives are great. Large-sized TiN are the main inclusions that affect the fatigue performance of 20CrMnTi. The average fatigue lives of 20CrMnTiCe exceed 10^7^ times, and the deviations of the fatigue lives are smaller than those of 20CrMnTi.

## 1. Introduction

20CrMnTi has good hardenability and high impact toughness at low temperatures and is widely used in the gear manufacturing industry [1,2,3,4]. Zhang et al. [5] have conducted an experimental study on the fatigue performance of 20CrMnTi and found that the fatigue life of 20CrMnTi is 3 × 10^6^ under the condition of experimental load of 100KN. Table 1 shows the inclusion size statistics in 20CrMnTi in the literature [6,7,8]. In order to improve the mechanical properties of 20CrMnTi, microalloying elements such as Ti are often added during smelting [9,10,11]. However, during the solidification of the molten steel, Ti inevitably combines with N to form TiN [7,12,13,14]. As TiN particles grow, they gradually develop into TiN inclusions with edges and corners. Moreover, such inclusions are difficult to eliminate during subsequent rolling and heat treatment processes, which can sharply deteriorate the toughness and fatigue resistance of the steel [15,16]. Therefore, studying the formations and modification mechanisms of TiN inclusions in steel is of great significance for improving the mechanical and service performance of 20CrMnTi.

Yan et al. [17] studied the precipitation behaviors of TiN in microalloyed steel and found that when the sizes of TiN particles precipitated were greater than 1 μm, they could be used as the starting positions of cracks and reduced the brittle transition temperature, which caused harm to the mechanical properties of the steel. Kim et al. [18] studied the thermodynamic equilibrium relationships between Ti and N contents and TiN formations in molten steel, indicating that the solubility of N in the pure iron solution increased with the increase in Ti content. When Ti and N contents exceeded their equilibrium concentration, pure solid TiN inclusions could precipitate from the molten steel. Yin et al. [19] studied the formation mechanisms of TiN inclusions in 17Cr austenitic stainless steel and found that there were high concentrations of Ti and N after feeding titanium wire, and TiN inclusions could precipitate in the molten steel, and the precipitated TiN could self-nucleate to form homogeneous TiN inclusions. Heterogeneous TiN inclusions could also be formed using existing Al_2_O_3_-MgO-TiO_x_ oxides as the cores. Ma et al. [20] studied the precipitations of TiN inclusions in the production of 20CrMnTi and found that significant increases in N contents were key factors leading to the precipitations of TiN inclusions. The smaller the contents of Ti and N in steel, the higher the cooling rates, and the smaller the sizes of TiN precipitated. Rare earth elements (RE) could effectively modify the shape and size of inclusions in steel, which was conducive to the improvement of the mechanical properties of steel. Yang et al. [21] studied the effects of RE on inclusions in high-carbon chromium-bearing steel and found that RE addition could modify irregular Al_2_O_3_ and MnS into relatively regular RE inclusions because of its active chemical property. Ahn et al. [22] studied the effects of Gd on the microstructures and mechanical properties of duplex stainless steels (DSS) and found that the oxygen content in the cast DSS alloy with Gd decreased because of the high reactivity of Gd with oxygen. The area fraction and size of non-intermetallic inclusions in the alloy decreased from 0.80 ± 0.12% to 0.58 ± 0.04% and from 6.9 ± 0.7 to 5.8 ± 0.4 μm upon Gd addition, respectively. Although many scholars have studied the formation mechanisms and growth behaviors of TiN inclusions [23,24,25,26], there are still few reports on the use of adding rare earth Ce to increase the nucleation cores in the molten steel of 20CrMnTi. Therefore, under rare earth Ce addition conditions, studying the formation mechanisms and controlling the sizes of TiN inclusions are of great significance for improving the mechanical performance of 20CrMnTi.

The addition of rare earth Ce is conducive to the formation of smaller nucleation cores in the steel molten pools, reducing the possibility of TiN aggregation and growth. The formed TiN inclusion size is smaller, which is more conducive to the improvement of steel fatigue properties. This paper uses theoretical calculations and experimental methods to study the formation mechanisms of TiN inclusions in 20CrMnTi under the condition of rare earth Ce addition. The appearances, quantities, sizes, and phase compositions of the composite inclusions containing TiN in the steels are analyzed statistically. The scientific natures of the reaction processes and interactions of Ti, N, Mg, Al, Ce, and O forming composite inclusions containing TiN are explored. This lays a theoretical foundation for the stable, high-quality, and efficient production of high-performance 20CrMnTi.

## 2. Materials and Methods

In order to explore the modification behaviors of TiN inclusions in gear steel before and after adding rare earth Ce, 20CrMnTi and 20CrMnTiCe were smelted separately. The chemical compositions of the two steels are shown in Table 2. Ce was determined by an inductively coupled plasma mass spectrometer (NexION 1000, Bao gang Group, Baotou, China), and C and S were determined by a carbon sulfur analyzer (EMIA-Pro, Bao gang Group, Baotou, China). Other elements were measured by an inductively coupled plasma spectrometer (5800 ICP-OES, Bao gang Group, Baotou, China). Both steels were smelted in a vacuum induction furnace in the laboratory. In order to ensure good deoxidation effects, Mg deoxidation was used during the smelting process. The molten steels were cast into 250 mm × 150 mm × 80 mm ingots. The ingots were heated to 1200 °C in the muffle furnace and kept for 240 min, and they were then rolled into 40 mm × 40 mm × 1500 mm rectangular billets. The billets were held at 850 °C for 20 min and then quenched in room temperature water. The quenched billets were then reheated to 180 °C and held for 80 min for low-temperature tempering.

**Figure 1:**
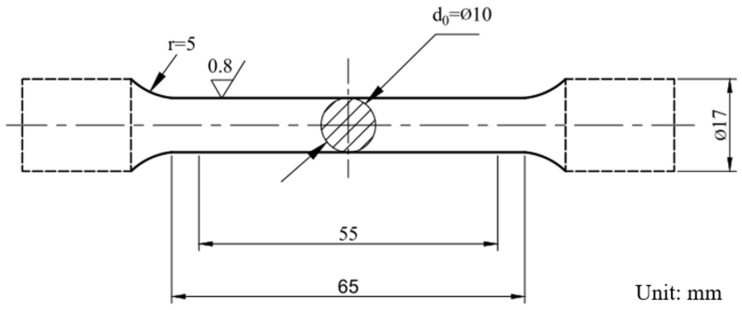
The geometry and dimensions of the fatigue specimen.

Thermo-Calc thermodynamic software (Thermo-Calc 2022a) was used to calculate the precipitations in the solidifications of 20CrMnTi. To evaluate the modification effects of rare earth Ce on TiN inclusions in the steels, metallographic samples were taken perpendicular to the rolling direction, and the corresponding inclusion parameters related to performance, such as inclusion types, sizes, and distributions, were studied by SEM (ZEISS, Baotou, China), EDS (Oxford X-MaxN20, Baotou, China), and EBSD (Oxford C Nano, Baotou, China). The size of the fatigue specimen is shown in Figure 1. According to GB/T3075-2021, the tests were conducted on a POWER SWING MOT 100 kN (Bao Gang Group, Baotou, China), with a stress ratio of R = −1, a load of 750 MPa, and a resonance frequency of 75 Hz. During the fatigue experiments, 8 samples were used to test both 20CrMnTi and 20CrMnTiCe. After the fatigue tests, SEM was used to analyze the fracture surfaces of the failed specimens, and EDS was used to analyze and evaluate the impacts of TiN inclusions with different shapes and compositions on the fatigue lives.

## 3. Results and Discussion

### 3.1. Morphologies and Compositions of the Inclusions Containing TiN in 20CrMnTi

**Figure 2:**
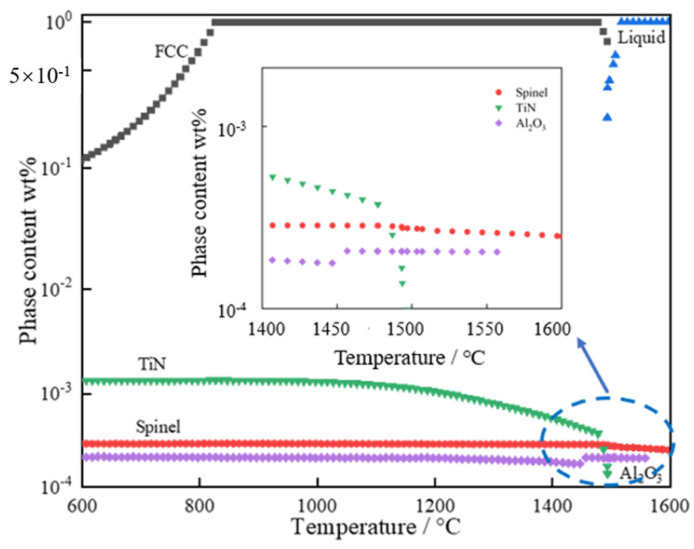
Thermodynamic calculations of precipitates during steel solidifications of 20CrMnTi.

Figure 2 shows the theoretical calculation results of material precipitation behaviors during equilibrium solidifications of 20CrMnTi. For 20CrMnTi, spinel and Al_2_O_3_ phases had already precipitated during the steelmaking process. The contents of spinel and Al_2_O_3_ phases tended to balance with the decrease in temperature. During the solidification process, TiN was generated, and its precipitation temperature was 1473 °C. Subsequently, its phase content did not change with the decrease in temperature. So, the spinel phase and Al_2_O_3_ formed earlier than TiN, providing the possibility for TiN to adhere to spinel and Al_2_O_3_ to grow.

**Figure 3:**
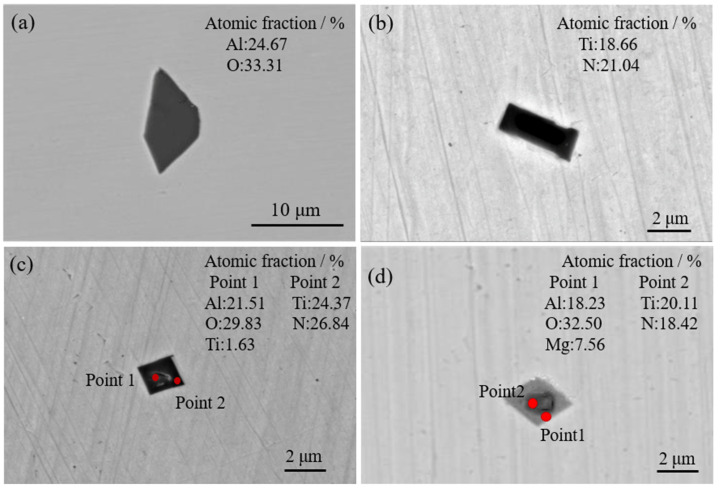
Morphologies and elements analysis of the typical inclusions in 20CrMnTi. (**a**,**b**) are single inclusions. (**c**,**d**) are complex inclusions.

**Figure 4:**
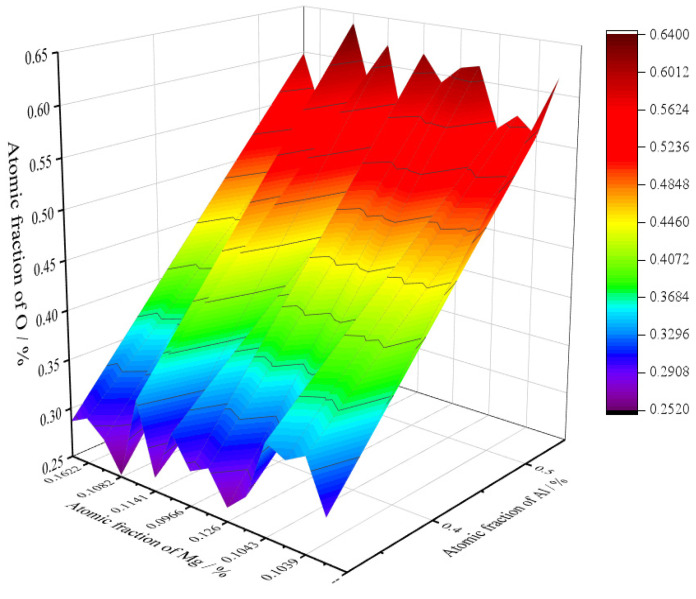
Atomic compositions of Mg-Al-O composite phase encapsulated with TiN.

Figure 3 shows the morphologies and elements distributions of the typical inclusions in 20CrMnTi. In Figure 3a,b, both were single inclusions, namely Al_2_O_3_ and TiN inclusions, with their sizes concentrated between 4 and 10 μm. In Figure 3c,d, both were composite inclusions, and the sizes were concentrated between 2 and 5 μm. The inclusion in Figure 3c was an Al_2_O_3_-coated TiN inclusion. The inclusion in Figure 3d was an Mg-Al-O-coated TiN composite phase. The inclusions containing TiN in 20CrMnTi were mostly square or diamond-shaped. In order to determine the core composition of the composite inclusion in Figure 3d, the numbers of the atoms of the main elements in the core were normalized, as shown in Figure 4. The horizontal and vertical coordinates of Figure 4 are the atomic fractions of Mg, Al, and O, respectively. The atomic number ratio of Mg-Al-O was 1:2.7:5.2, so the composite inclusion was aluminum–magnesium spinel (MgAlO_4_), which was consistent with thermodynamic calculations.

**Figure 5:**
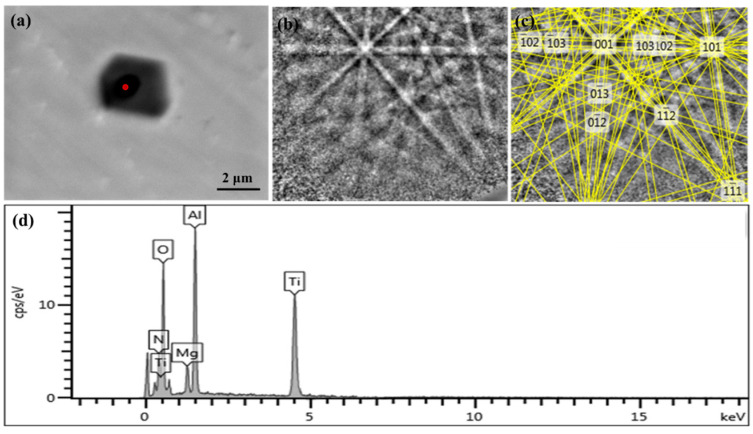
Identification of MgAlO_4_ phase. (**a**) morphology of the inclusion, (**b**) Kikuchi pattern of the inclusion nucleation core, (**c**) Kikuchi pattern with nucleation core crystal orientation, (**d**) the distribution of element types.

In order to investigate the compositions of the nucleation cores of the typical inclusions containing TiN in 20CrMnTi, SEM, EBSD, and EDS were used. The Mg-Al-O composite phase in the typical nucleating core was searched, and the specific phase of Mg, Al, and O was accurately obtained. The Kikuchi pattern and energy spectrum were obtained, as shown in Figure 5. Figure 5a shows the inclusion morphology, and Figure 5b shows the Kikuchi pattern of the inclusion nucleation core, which can be used to analyze the crystal orientation of the nucleation core. Figure 5c shows the Kikuchi pattern with nucleation core crystal orientation marked in Figure 5b. Figure 5d shows the distribution of element types in the nucleation core and surrounding materials of the inclusion. The main elements of the nucleating core in the composite phase were Ti, N, Mg, Al, and O. Through Kikuchi pattern calibration of EBSD, the composite phase was MgAlO_4_. This was consistent with the analysis of the element compositions in the energy spectrum, indicating that MgAlO4 could be used as the nucleation core of TiN in 20CrMnTi.

### 3.2. Morphologies and Compositions of the Inclusions Containing TiN in 20CrMnTiCe

**Figure 6:**
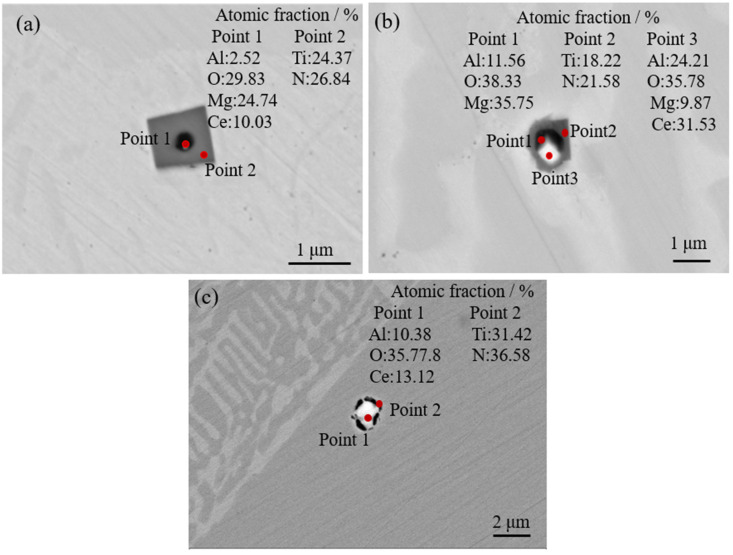
Morphologies and elements analysis of the typical inclusions in 20CrMnTiCe. (**a**–**c**) are typical complex inclusions in 20CrMnTiCe.

Figure 6 shows the morphologies and compositions of the inclusions in 20CrMnTiCe. The sizes of the inclusions were all below 2 μm. Compared with the inclusion sizes in 20CrMnTi, the addition of Ce could effectively reduce the inclusion sizes. After the addition of Ce, the material formed in the center of the inclusion contained Ce element, and TiN was wrapped around it to form a composite inclusion. In the process of steel smelting, because the raw material contained Al, it was difficult to completely remove Al from the experimental steels. Therefore, regardless of any deoxidation method, the steels still retained 0.01% Al. Al was bound to participate in forming the nucleation core. As shown in Figure 6a,b, Mg-Al-O-Ce composite phase was used as the nucleation core. Therefore, Mg-Al-O-Ce and Al-O-Ce nucleation cores appeared in steel, and these two types of composite phases could become high-quality nucleation cores for TiN inclusions.

**Figure 7:**
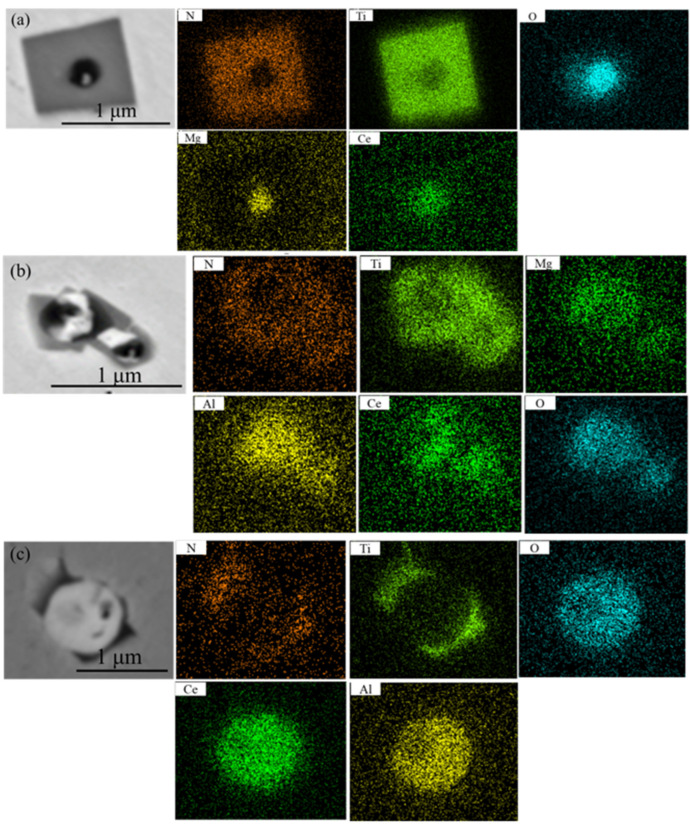
The element distributions of the typical inclusions in 20CrMnTiCe. (**a**–**c**) are the element distributions of the different typical complex inclusions.

Figure 7 shows the distributions of the typical inclusion elements in 20CrMnTiCe. The elements of Ti and N were mainly distributed in the periphery, and the cores of the inclusions were the composite phases formed by the elements of Al, Mg, O, and Ce. The addition of Ce element was conducive to the formation of the inclusion core, and TiN inclusions precipitated in steel would gradually grow up with this core.

**Figure 8:**
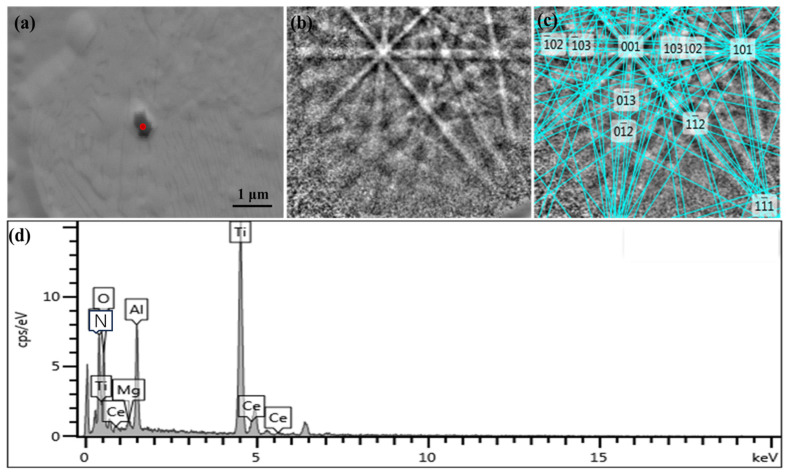
Identification of MgAlO_4_-Ce phase. (**a**) morphology of the inclusion, (**b**) Kikuchi pattern of the inclusion nucleation core, (**c**) Kikuchi pattern with nucleation core crystal orientation, (**d**) the distribution of element types.

**Figure 9:**
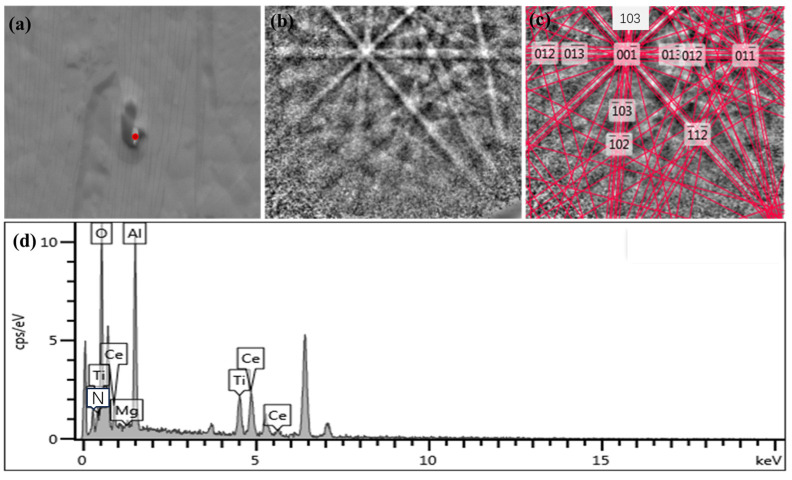
Identification of CeAlO_3_ phase. (**a**) morphology of the inclusion, (**b**) Kikuchi pattern of the inclusion nucleation core, (**c**) Kikuchi pattern with nucleation core crystal orientation, (**d**) the distribution of element types.

By means of SEM, EBSD, and EDS, all the composite phases containing Ce and TiN in 20CrMnTiCe were searched and identified, as shown in Figure 8 and Figure 9. Figure 8a and Figure 9a show the inclusion morphologies, and Figure 8b and Figure 9b show the Kikuchi patterns of the inclusion nucleation cores, which can be used to analyze the crystal orientations of the nucleation cores. Figure 8c and Figure 9c show the Kikuchi patterns with nucleation core crystal orientations marked in Figure 8b and Figure 9b. Figure 8d and Figure 9d show the distributions of element types in the nucleation cores and surrounding materials of the inclusions. Through the calibration of the Kikuchi pattern of the TiN nucleated core, combined with the element qualitative analysis of the energy spectrum, the core phases of TiN nucleation were MgAlO_4_-Ce and CeAlO_3_ phases. With the addition of Ce, MgAlO_4_-Ce and CeAlO_3_ phases could be used as nucleation cores for TiN inclusions. The content of Ti element in the steel was certain; therefore, compared with 20CrMnTi without Ce, the addition of Ce would increase the nucleation cores of TiN, avoiding the possibility of TiN aggregation and growth, so the formed TiN inclusion size was smaller.

### 3.3. Effects of Ce on the Quantities and Sizes of the Inclusions Containing TiN in Steels

**Figure 10:**
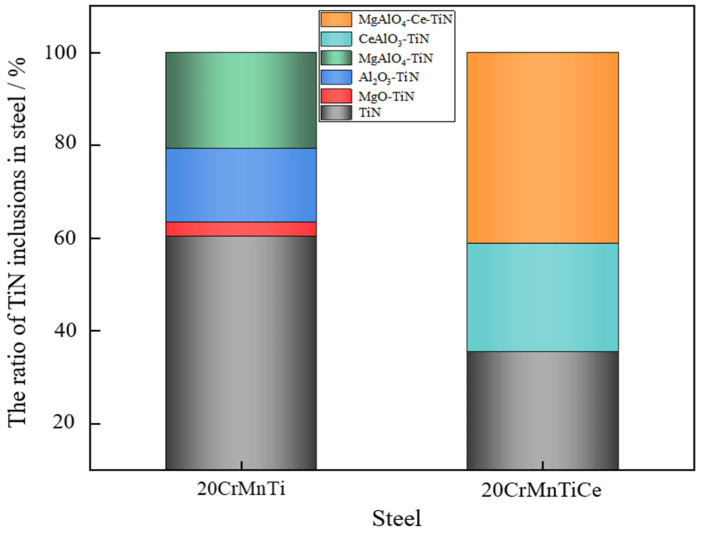
Statistics of titanium inclusions in steel.

The above research results showed that MgAlO_4_-Ce and CeAlO_3_ could be used as the nucleation cores of TiN to form smaller and spherical composite inclusions, which could serve the purpose of TiN modification. Therefore, in order to quantify the TiN modification effects, the types and proportions of the titanium inclusions before and after adding Ce were statistically analyzed, as shown in Figure 10. The proportion of the pure TiN inclusions in 20CrMnTiCe was about 60%, and that in 20CrMnTi was about 36.7%. After adding Ce, the proportions of the composite inclusions with MgAlO_4_-Ce and CeAlO_3_ as the cores increased. The decrease in the proportion of pure TiN inclusions prevented their excessive growth to some extent.

Table 3 shows the proportional distributions of TiN inclusion sizes before and after Ce addition. For the inclusions sized smaller than 2 μm, the proportion in 20CrMnTiCe was 76.6%, which was significantly increased compared with 45.9% in 20CrMnTi. The statistical analysis of 5–10 μm inclusions showed that the proportions of TiN inclusions decreased from 14.3% to 4.2% after adding Ce. Therefore, the addition of Ce could refine the sizes of TiN inclusions, which is beneficial for the improvement of the mechanical properties of the gear steel.

### 3.4. Effects of Ce on Fatigue Properties of the Steels

**Figure 11:**
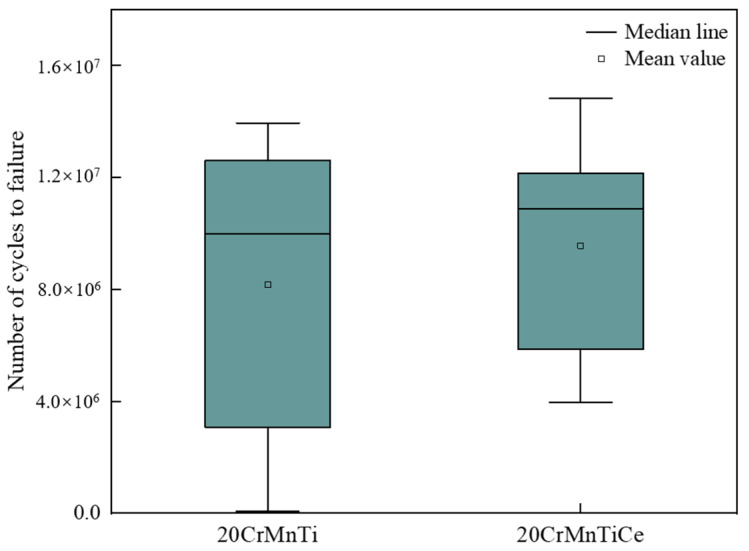
Fatigue lives of different samples.

**Figure 12:**
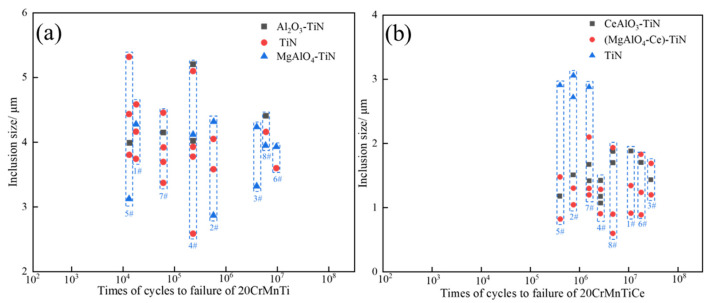
Statistics of inclusion sizes at different sample fractures. 8 samples were used to test both 20CrMnTi and 20CrMnTiCe, and they are numbered 1# to 8# in the two steels. (**a**) 20CrMnTi, (**b**) 20CrMnTiCe.

In order to verify whether the rare earth Ce addition was good or bad for the fatigue performance of the steels, fatigue tests were carried out. The test results are shown in Figure 11. The average fatigue lives of 20CrMnTi did not reach 10^7^ times, the deviations between the maximum and minimum fatigue lives were great, and the performance stabilities needed to be improved. After the addition of Ce, the fatigue strength of 20CrMnTiCe was significantly higher than that of 20CrMnTi, the average fatigue lives were more than 10^7^ times, and the fatigue life fluctuation was small, which met the performance requirements. The compositions of the inclusions at the fracture surfaces were analyzed. The statistical results are shown in Figure 12. In the fatigue fracture samples of 20CrMnTi, the main inclusions at the fractures of low-life regions were large-size TiN and Al_2_O_3_-TiN. In the samples of 20CrMnTiCe, the main inclusions at the fractures of the low-life regions were still large-size TiN, and (MgAlO_4_-Ce)-TiN and (CeAlO_3_)-TiN were distributed in both high- and low-life regions.

**Figure 13:**
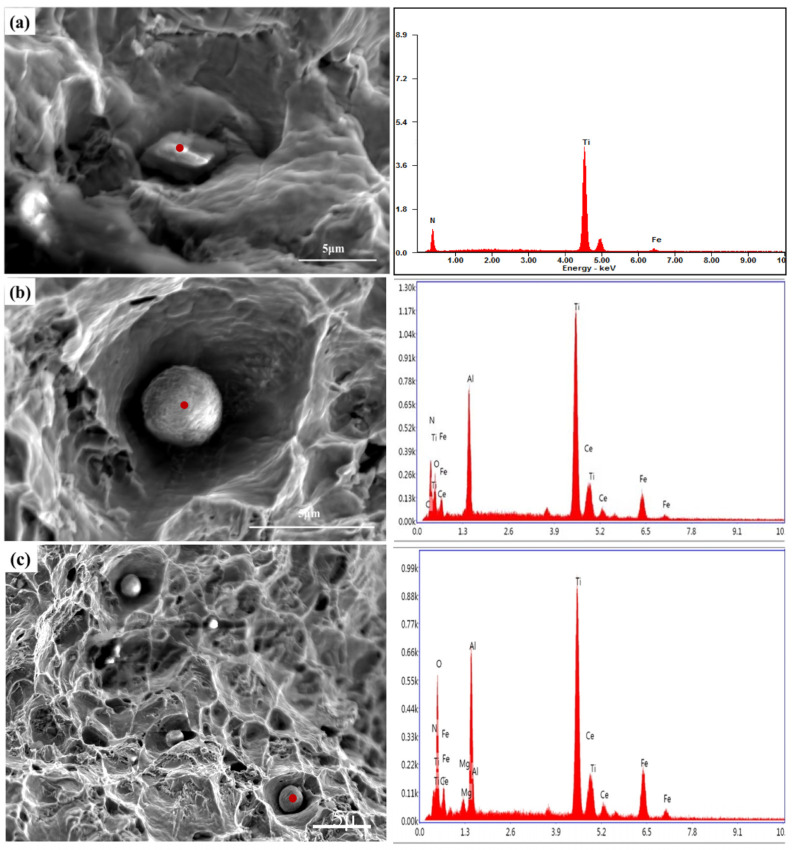
Morphologies of inclusions at the fracture surface of 20CrMnTi and 20CrMnTiCe. (**a**) TiN inclusion in 20CrMnTi, (**b**) CeAlO_3_-TiN inclusion in 20CrMnTiCe, (**c**) (MgAlO_4_-Ce)-TiN inclusion in 20CrMnTiCe.

Figure 13 shows the morphologies of the typical inclusions at the fracture surfaces in 20CrMnTi and 20CrMnTiCe. There were still square TiN inclusions in 20CrMnTi without Ce addition, as shown in Figure 13a. The addition of rare earth Ce was conducive to the formation of smaller, spherical nucleation cores, reducing the possibility of TiN aggregation and growth. Typical spherical inclusions were found in 20CrMnTiCe after Ce addition, as shown in Figure 13b,c. By analyzing the compositions of the inclusions at the fatigue fractures, it could be seen that the large-size TiN was the main inclusion affecting fatigue strength. The addition of Ce was beneficial to the formation of MgAlO_4_-Ce and CeAlO_3_ in TiN nucleation cores, and the formation of smaller (MgAlO_4_-Ce)-TiN and (CeAlO_3_)-TiN composite inclusions, thus improving the fatigue properties of the steels.

## 4. Conclusions

(1) The main inclusions in 20CrMnTi are TiN, Al_2_O_3_, Al_2_O_3_-TiN, and MgAlO_4_-TiN, among which the inclusions containing TiN are mostly square or diamond-shaped, and the pure TiN inclusions account for 60% of the total inclusions. After the addition of rare earth Ce, the main titanium inclusions of 20CrMnTiCe are TiN, MgAlO_4_-TiN, and CeAlO_3_-TiN, among which pure TiN inclusions account for 36.7% of the total inclusions. The generated MgAlO_4_-TiN and CeAlO_3_-TiN are spherical, indicating that rare earth Ce can modify some TiN inclusions.
(2) The sizes of Al_2_O_3_ and TiN in 20CrMnTi are concentrated in 2–5 μm. The composite inclusions of Al_2_O_3_-TiN and MgAlO_4_-TiN are concentrated in sizes ranging from 4 to 10 μm, and their proportion is 14.3%. After adding rare earth Ce, the sizes of MgAlO_4_-TiN and CeAlO_3_-TiN less than 2 μm accounted for 63.3%. Rare earth Ce can refine the inclusions containing TiN in 20CrMnTi.
(3) The average fatigue lives of 20CrMnTi do not reach 10^7^ times, and the deviations between the maximum and minimum fatigue lives are great, while large-sized TiN is the main inclusion affecting the fatigue performance. The addition of Ce can modify TiN inclusions, forming spherical and small-sized composite inclusions of (MgAlO_4_Ce)-TiN and (CeAlO_3_)-TiN. The average fatigue lives exceed 10^7^ times, and the deviations of the fatigue lives are smaller than those of 20CrMnTi.

## Figures and Tables

**Table 1:** Inclusion size statistics in 20CrMnTi in the literature [6,7,8].

Literature	<5 μm	>5 μm
Li et al. [6]	main	many
Wang et al. [7]	74.11%	25.89%
Fang et al. [8]	average size ≤ 5 μm	

**Table 2:** Chemical compositions of the steels.

Samples	C	Si	Mn	Al	Cr	Ti	Mg	O	N	Ce	Fe
	(wt/%)							(ppm)			
20CrMnTi	0.24	0.18	0.95	0.011	1.02	0.052	138	44	50	/	Bal.
20CrMnTi-Ce	0.25	0.20	0.93	0.010	1.00	0.054	130	33	47	62	Bal.

**Table 3:** The proportional distributions of the inclusion sizes.

Steel	<2 μm	2–5 μm	5–10 μm
	Proportion/%		
20CrMnTi	45.9	39.8	14.3
20CrMnTiCe	76.6	19.2	4.2

## Data Availability

Not applicable.

## References

[1] Zhang X., Tang J.Y. (2017). Effect of 20CrMnTi material compositions fluctuation on gear performance after carburizing and quenching process. J. Cent. South Univ..

[2] Wang X., Wei X., Yang X., Cheng Z., Wang W. (2013). Atomic diffusion of gradient ultrafine structured surface layer produced by T10 steel rubbing against 20CrMnTi steel. Wear.

[3] Zhu Y., Lu Y.-M., Huang C.-W., Liang Y.-L. (2020). The effect of TiN inclusions on the fracture mechanism of 20CrMnTi steel with lath martensite. Mater. Res. Express.

[4] Li Y., Cheng G., Lu J., Sun J. (2022). Characteristics and Formation Mechanism of Complex TiN Inclusions in 20CrMnTi Gear Steel. ISIJ Int..

[5] Zhang M., Wang Z., Feng X.L., Xie L.Z., Liu A.J., Liu N. (2017). Research on fatigue of 20CrMnTi steel gear. Heat Treat..

[6] Li Y., Cheng G.G., Lu J.L., Sun J. (2020). Characteristics and distribution of TiN inclusions in 20CrMnTi gear steel. China Metall..

[7] Wang X.Y. (2018). Study on Control of Non-Metallic Inclusions in 20CrMnTi Gear Steel.

[8] Fang Y.R., Wang C.R., Xu Y.H., Hu Y.J., Zhan D.P. (2021). Research on inclusions in billet of 20CrMnTiH gear steel. J. Liaoning Inst. Sci. Technol..

[9] Tang J., Hu X., Lai F., Guo X., Qu S., He R., Qin S., Li J. (2020). Evolution of Fretting Wear Behaviors and Mechanisms of 20CrMnTi Steel after Carburizing. Metals.

[10] Wang J.B., Ding J., Song K., Zhang X.D., Wang L.S. (2019). Dynamic Stress Response of 20CrMnTi Steel under Impact Load with Modified J-C Ontogenetic Model Considering Adiabatic Temperature Rise. Mech. Strength.

[11] Wang D.K. (2020). Research on Flow Behaviors and Microstructure Evolutions during Warm/Hot Deformation in 20CrMnTi Low-Alloy Steel.

[12] Yuan S., Liang G. (2009). Dissolving behaviour of second phase particles in Nb–Ti microalloyed steel. Mater. Lett..

[13] Shi Z., Wang R., Hang S., Feng C., Wang Q., Yang C. (2016). Effect of nitrogen content on the second phase particles in V–Ti microalloyed shipbuilding steel during weld thermal cycling. Mater. Des..

[14] Wang J., Peng J., Zhang F., Li Y., Zhang X., An S. (2023). Effects of Ce-Modified TiN Inclusions on the Fatigue Properties of Gear Steel 20CrMnTi. Crystals.

[15] Cheng L. (2020). Study on Evolution and Control of Inclusions in 20CrMnTi Gear Steel.

[16] Qi H.Q. (2010). Study on the Effect of Composition Optimization of 20CrMnTi Steel on Microstructure and Contact Fatigue Life.

[17] Yan W., Shan Y.Y., Yang K. (2006). Effect of TiN inclusions on the impact toughness of low-carbon microalloyed steels. Met. Mater. Trans. A.

[18] Kim W.-Y., Jo J.-O., Chung T.-I., Kim D.-S., Pak J.-J. (2007). Thermodynamics of Titanium, Nitrogen and TiN Formation in Liquid Iron. ISIJ Int..

[19] Yin X., Sun Y., Yang Y., Bai X., Barati M., Mclean A. (2016). Formation of Inclusions in Ti-Stabilized 17Cr Austenitic Stainless Steel. Met. Mater. Trans. B.

[20] Ma W.-J., Bao Y.-P., Zhao L.-H., Wang M. (2014). Control of the precipitation of TiN inclusions in gear steels. Int. J. Miner. Met. Mater..

[21] Yang C., Luan Y., Li D., Li Y. (2019). Effects of rare earth elements on inclusions and impact toughness of high-carbon chromium bearing steel. J. Mater. Sci. Technol..

[22] Ahn J.-H., Jung H.-D., Im J.-H., Jung K.H., Moon B.-M. (2016). Influence of the addition of gadolinium on the microstructure and mechanical properties of duplex stainless steel. Mater. Sci. Eng. A.

[23] Zhou D.G., Jie F.U., Chen X.C. (2003). Precipitation behavior of TiN in bearingsteel. J. Mater. Sci. Technol..

[24] Yin X., Sun Y., Yang Y., Deng X., Barati M., McLean A. (2016). Effect of alloy addition on inclusion evolution in stainless steels. Ironmak. Steelmak..

[25] Liu H.Y., Wang H.L., Li L., Zheng J.Q., Li Y.H., Zeng X.Y. (2011). Investigation of Ti inclusions in wire cord steel. Ironmak. Steelmak..

[26] Qu T.-P., Tian J., Chen K.-L., Xu Z., Wang D.-Y. (2017). Precipitation behaviour of TiN in Nb-Ti containing alloyed steel during the solidification process. Ironmak. Steelmak..

